# RAD sequencing sheds new light on the genetic structure and local adaptation of European scallops and resolves their demographic histories

**DOI:** 10.1038/s41598-019-43939-4

**Published:** 2019-05-15

**Authors:** David L. J. Vendrami, Michele De Noia, Luca Telesca, William Handal, Grégory Charrier, Pierre Boudry, Luke Eberhart-Phillips, Joseph I. Hoffman

**Affiliations:** 1 Department of Animal Behavior, University of Bielefeld, Postfach 100131, 33615 Bielefeld, Germany; 2 Institute of Biodiversity, Animal Health & Comparative Medicine, College of Medical, Veterinary & Life Sciences, University of Glasgow, Glasgow, UK; 3 Department of Earth Sciences, University of Cambridge, Downing Street, Cambridge, CB2 3EQ United Kingdom; 4 British Antarctic Survey, High Cross, Madingley Road, Cambridge, CB3 OET United Kingdom; 5 University of Brest, Laboratoire des Sciences de l’Environnement Marin (LEMAR, UMR 6539, UBO/CNRS/IRD/Ifremer), European University Institute for the Sea (IUEM), rue Dumont d’Urville, 29280 Plouzané, France; 6 Ifremer, Laboratoire des Sciences de l’Environnement Marin (UBO/CNRS/IRD/Ifremer), Plouzané, France; 7 Department of Evolutionary Ecology and Behavioural Genetics, Max Planck Institute for Ornithology, Seewiesen, Germany

**Keywords:** Molecular ecology, Population genetics

## Abstract

Recent developments in genomics are advancing our understanding of the processes shaping population structure in wild organisms. In particular, reduced representation sequencing has facilitated the generation of dense genetic marker datasets that provide greater power for resolving population structure, investigating the role of selection and reconstructing demographic histories. We therefore used RAD sequencing to study the great scallop *Pecten maximus* and its sister species *P. jacobeus* along a latitudinal cline in Europe. Analysis of 219 samples genotyped at 82,439 single nucleotide polymorphisms clearly resolved an Atlantic and a Norwegian group within *P. maximus* as well as *P. jacobeus*, in support of previous studies. Fine-scale structure was also detected, including pronounced differences involving Mulroy Bay in Ireland, where scallops are commercially cultured. Furthermore, we identified a suite of 279 environmentally associated loci that resolved a contrasting phylogenetic pattern to the remaining neutral loci, consistent with ecologically mediated divergence. Finally, demographic inference provided support for the two *P. maximus* groups having diverged during the last glacial maximum and subsequently expanded, whereas *P. jacobeus* diverged around 95,000 generations ago and experienced less pronounced expansion. Our results provide an integrative perspective on the factors shaping genome-wide differentiation in a commercially important marine invertebrate.

## Introduction

Over the last decade, the development of genotyping approaches based on reduced representation sequencing has led to a step change in our understanding of the population genetics of wild organisms^1^. In particular, restriction site associated DNA (RAD) sequencing has increased in popularity due to its ability to generate large single nucleotide polymorphism (SNP) datasets even for species lacking genomic resources^2^. These datasets not only provide greater power to resolve genetic differences among populations, but also allow the underlying drivers of population structure and genetic diversity to be investigated^3^. Of these drivers, neutral processes such as allopatric divergence and effective population size change are expected to play a prominent role, particularly in marine species that have experienced profound changes in habitat availability in relation to glacial cycles^4^. However, the widespread discovery of environmentally associated loci^5^ has also pointed towards an important contribution of non-neutral processes towards observed patterns of genome-wide divergence. Investigating the contributions of these different processes offers the opportunity to better understand contemporary patterns of population structure and genetic diversity.

The great scallop *Pecten maximus* provides an interesting case study. This marine invertebrate can be found widely along the European Atlantic seaboard and is economically important, with annual catches exceeding 55,000 tonnes (FAO, http://www.fao.org/fishery/species/3516/en). Despite being able to swim for short distances by rapidly clapping its valves when escaping predators^6^, *P. maximus* is generally considered to be sedentary at the adult stage. However, the potential of this species to disperse is believed to be considerable, as it possesses planktonic larvae that are capable of drifting in the water column from 18 to 42 days^7^ and *P. maximus* is also capable of secondary dispersal via byssus drifting^8^.

Several studies have used genetic analysis to investigate the population structure of *P. maximus* in European coastal waters. Studies based on allozymes and mitochondrial DNA did not identify any genetic differences between French and British populations^9–11^ but significant mitochondrial haplotype frequency differences were found between the UK and Norway^12^. These patterns were subsequently confirmed by Morvezen *et al*.^13^ who combined an extensive sample set of populations ranging from Spain to Norway with microsatellites to provide a more detailed nuclear perspective. They found evidence for two distinct genetic clusters, an ‘Atlantic group’ comprising populations from the Galician coast to the Irish Sea, and a ‘Norwegian group’ comprising scallops sampled along the Norwegian coast. However, two main questions relating to the origin of the two groups remain to be resolved.

First, it is unclear whether the Norwegian group is restricted to Norway. This would be expected if the Norwegian trench^14^ acts as the primary barrier to gene flow between the two *P. maximus* lineages, as has been observed in many other benthic macrofaunal species^15^. Alternatively, hydrographic features of the North Sea^16^ could potentially restrict gene flow but nevertheless allow secondary contact and local mixing between the two lineages. To investigate this, analysis of additional samples from around the UK and in particular from the North Sea is required to better define the geographical boundaries of the two genetic groups. Second, it is not known yet if the Atlantic and Norwegian groups originated from a single glacial refugium in Southern Europe or whether they persisted during the Last Glacial Maximum (LGM) in two separate refugia, one in Southern Europe and one in Northern Europe. Morvezen *et al*.^13^ did not formally test these alternative hypotheses, although they reported a decline in microsatellite diversity with increasing latitude that might be consistent with expansion from a single refugium followed by sequential founder effects as the species spread northwards. To evaluate which hypothesis is most likely, dense genetic marker data subjected to demographic modelling could be used to gauge support for alternative models capturing the main properties of these contrasting historical scenarios.

A further topic that remains to be investigated in European scallops is local adaptation, which is also amenable to analysis using dense genetic marker data. Broadly, there is a large body of evidence suggesting that local adaptation to temperature is a pervasive phenomenon across marine invertebrates^17^. More specifically, a related scallop species (*Placopecten magellanicus*) in North America was found to exhibit geographical variation in thermal tolerance^18^ as well as significant genetic associations with sea surface temperature (SST), implying an important role of ecologically mediated divergence^19^. In European scallops, growth performance correlates positively with SST^20^ and proteomic differences have also been observed between scallops from different latitudes^21^, although it is not known if these differences have a mainly genetic or plastic basis. By contrast, common garden experiments suggest that naturally observed latitudinal variation in reproductive traits in *P. maximus* has a genetic basis^22^. Consequently, some aspects of physiological performance may be dependent on locally adapted genetic architectures that in principle could be inferred from genotype‒environment associations. Moreover, emerging approaches based on machine learning regression tree based algorithms, such as gradient forest analysis^23^, can provide a more nuanced understanding of which environmental factors best explain genetic variation across an environmental gradient and may therefore highlight the importance of other variables in addition to SST in shaping local adaptation^24^.

Finally, *P. maximus* also has a sister species, *P. jacobeus*, which is present in the Mediterranean basin. These taxa therefore represent an example of closely related species pairs that replace each other in the Mediterranean and along the Atlantic coast, similar to the limpets *Patella caerulea* and *P. depressa*^25^ and the crabs *Carcinus aestuarii* and *C. maenas*^26^. It was originally thought that the two *Pecten* species shared a common ancestor around five million years ago and diverged shortly after the Messinian Salinity Crisis when the Atlantic Ocean reclaimed the Mediterranean basin^27^. However, this view has been challenged by studies using allozymes^28^, mitochondrial DNA^29,30^ and microsatellites^13^, all of which found lower than expected differentiation. The divergence time of *P. maximus and P. jacobeus* also appears to be more recent than expected, although current estimates have a high degree of uncertainty^30,31^. Dense SNP data may therefore be able to shed new light on this topic by allowing more precise estimation of both the magnitude of genetic differentiation and the most likely divergence time.

**Figure 1:**
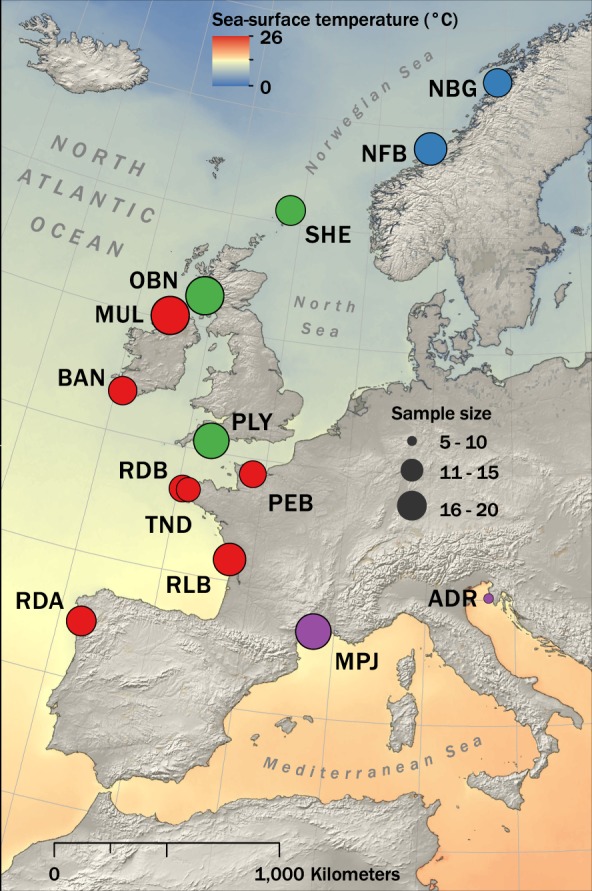
Map showing scallop sampling locations against a backdrop of variation in mean annual sea surface temperature. Blue circles represent populations from the Norwegian *P. maximus* group defined by Morvezen *et al.*^13^. Populations from the Atlantic group defined by Morvezen *et al.*^13^ are subdivided for ease of interpretation into a southern Atlantic group (color coded in red) and a northern Atlantic group (color coded in green) as described in the Results. Purple circles represent *P. jacobeus* populations from the Mediterranean Sea. The oceanic raster visualizes a composite of annual mean sea-surface temperatures (°C) measured from the Aqua Modis satellite between 2009 and 2013. The map was generated using the program ArcGIS 10.1 (ESRI, Redlands, CA, USA; https://www.esri.com/en-us/arcgis) using public domain vector and raster spatial data from naturalearthdata.com.

In order to address the above questions, we used RAD sequencing to genotype a total of 240 *P. maximus* and 40 *P. jacobeus* samples collected across 14 sites encompassing a latitudinal European cline from Spain to northern Norway (Fig. 1 and Table 1). We characterized population structure and used coalescent modeling to infer divergence times between the major European scallop lineages. We also investigated patterns of genetic diversity and their relationship to effective population size variation and sought to identify loci associated with environmental factors as well as to identify environmentally mediated population structure.

**Table 1:** Table of sampling locations showing population identity and location, species identity (*P. maximus* or *P. jacobeus*) and the number of samples that were retained for analysis after quality control and filtering.

Population ID	Location	Species	Samples passing QC	MLH (mean, SE)	NPL (mean, SE)	Observed number of polymorphic loci
RDA	Moaña, Spain	*P. maximus*	16	0.132 (0.001)	30,853.95 (259.16)	53,621
MPJ	Lion Gulf, France	*P. jacobeus*	19	0.123 (0.0005)	30,062.93 (194.14)	52,452
ADR	Novigrad Bay and Banjole, Croatia	*P. jacobeus*	5	0.118 (0.002)	24,407.35 (324.96)	30,644
RLB	La Tremblade, France	*P. maximus*	17	0.133 (0.001)	31,627.37 (276.89)	55,733
RDB	Bay of Brest, France	*P. maximus*	14	0.133 (0.001)	30,997 (236.42)	51,634
TND	Tinduff, France	*P. maximus*	13	0.149 (0.003)	30,506 (239.24)	48,692
PEB	Port-en-Bessin, France	*P. maximus*	14	0.133 (0.001)	29,866 (258.24)	50,285
PLY	Tucker Rock, England	*P. maximus*	19	0.137 (0.002)	31.854 (224.09)	57,396
BAN	Bantry Bay, Ireland	*P. maximus*	15	0.191 (0.004)	30,271 (261.56)	51,044
MUL	Mulroy Bay, Northern Ireland	*P. maximus*	20	0.131 (0.001)	31,706.16 (265.46)	52,811
OBN	Oban, Scotland	*P. maximus*	19	0.133 (0.001)	31,844 (254.81)	57,751
SHE	Fetlar Island, Scotland	*P. maximus*	16	0.131 (0.001)	28,797.11 (241.34)	51,854
NFB	Froan, Norway	*P. maximus*	17	0.131 (0.001)	28,233.57 (236.24)	46,518
NBG	Bodø, Norway	*P. maximus*	15	0.129 (0.0005)	27,985.58 (223.51)	45,539

Two genetic diversity statistics are also provided. Heterozygosity is given as mean +/− SE multilocus heterozygosity (MLH) while the mean +/− SE number of polymorphic loci (NLP) is given based on 1,000 randomized datasets of five individuals per population (see Materials and Methods for details). Population specific total numbers of polymorphic loci not corrected for sample size are also shown.

## Results

To evaluate population structure, historical demography and local adaptation in European scallops, we RAD sequenced a representative sample of *P. maximus* and *P. jacobeus* individuals. The sampling sites comprised (i) two *P. maximus* populations from the Norwegian group (blue circles in Fig. 1); (ii) ten *P. maximus* populations from the Atlantic group, which were further subdivided on the basis of *post hoc* analyses into the northern and southern Atlantic groups (green and red circles respectively in Fig. 1); and (iii) two *P. jacobeus* populations (purple circles in Fig. 1). A total of 823,811,871 50 bp sequence reads were generated and *de novo* assembled into 606,048 loci, from which we called 564,526 raw SNPs. Application of the filtering criteria described in the Materials and Methods and summarized in Supplementary Table 1 resulted in a final dataset of 219 samples genotyped at 82,439 high quality SNPs. The mean depth of coverage over all retained SNPs was 16.07, with the upper and lower 5% quantiles being 12.76 and 23.88 respectively.

### Broad scale patterns of population genetic differentiation

To formally test for genetic differences, we calculated pairwise *F*_st_ values among all of the sampling localities and determined their statistical significance through permutation (Supplementary Table 2). The highest *F*_st_ values were obtained for comparisons involving *P. maximus* and *P. jacobeus* (*F*_st_ = 0.03–0.09, *p* < 0.01). Within *P. maximus*, most of the comparisons involving populations of the Norwegian and Atlantic groups yielded somewhat smaller but significant *F*_st_ values (*F*_st_ = 0.02–0.05, *p* < 0.01). Furthermore, a number of comparisons within the Atlantic group, and particularly involving Mulroy Bay, were significant (*F*_st_ = 0.0001–0.02, *p* < 0.02), indicating the presence of subtle fine-scale structuring.

**Figure 2:**
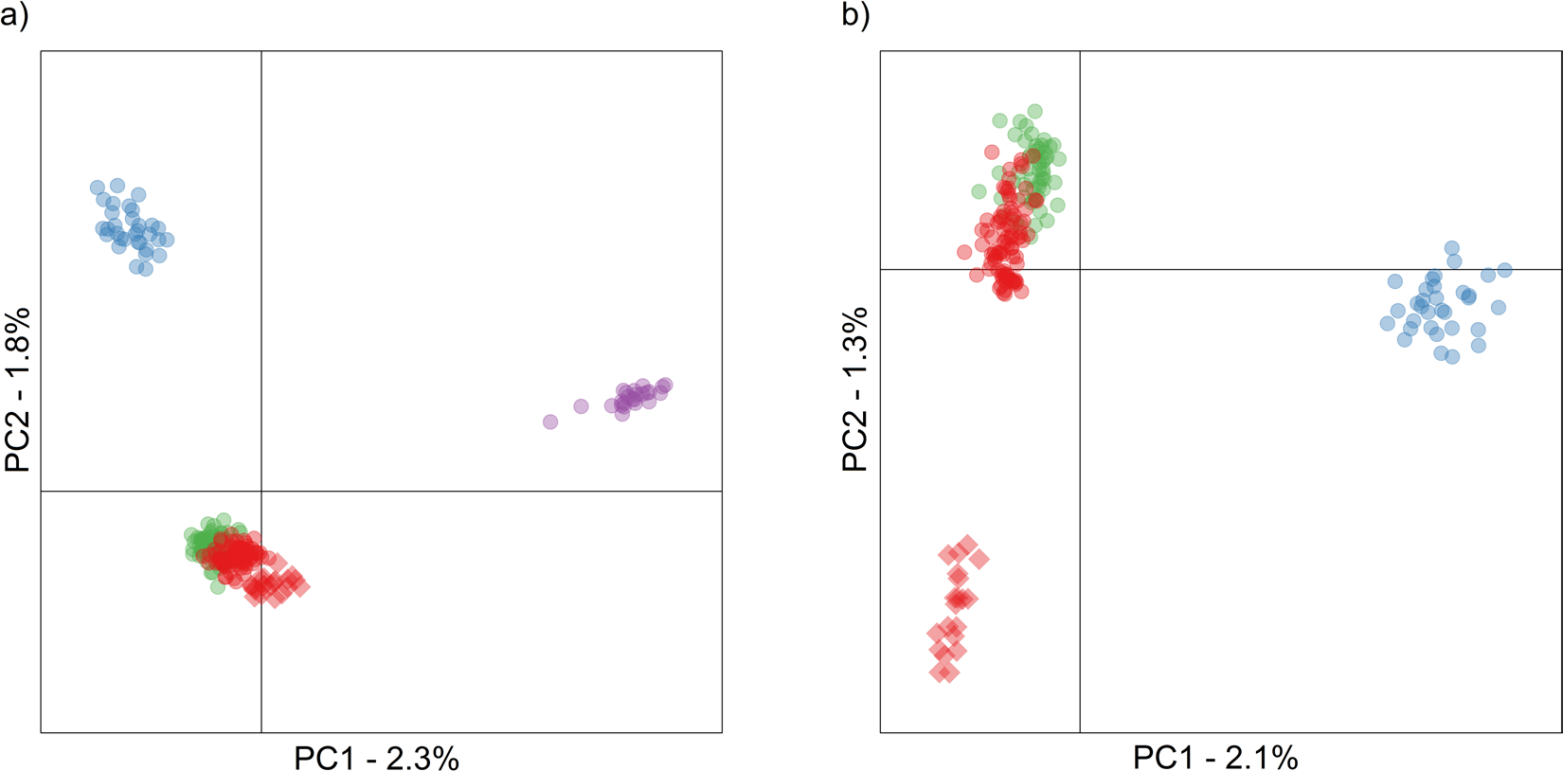
Scatterplot showing individual variation in principal component (PC) scores derived from principal component analysis (PCA) of the genomic data. Panels (a) and (b) show results including and excluding the Mediterranean populations respectively. The amounts of variation explained by each PC are given as percentages. Samples are color coded as described in the legend of Fig. 1 and scallops from Mulroy Bay are indicated by diamonds.

To evaluate genetic structure at the individual level, we performed a principal component analysis (PCA) of the full dataset (Fig. 2a). *P. maximus* clearly separated apart from *P. jacobeus* along PC1, while the Norwegian and Atlantic *P. maximus* groups also separated apart from one another along PC2. Furthermore, *P. maximus* individuals from Scotland and the Shetland Islands clustered together with the Atlantic group, suggesting that the Norwegian group may consist exclusively of scallops from Norway. Restricting the analysis to *P. maximus* confirmed this pattern while also emphasizing two further aspects (Fig. 2b). First, scallops from Mulroy Bay separated apart from the rest of the Atlantic group, in line with a recent fine-scale study in Northern Ireland^32^. Second, there was also a weak tendency for the northern and southern Atlantic samples to separate along PC2.

**Figure 3:**
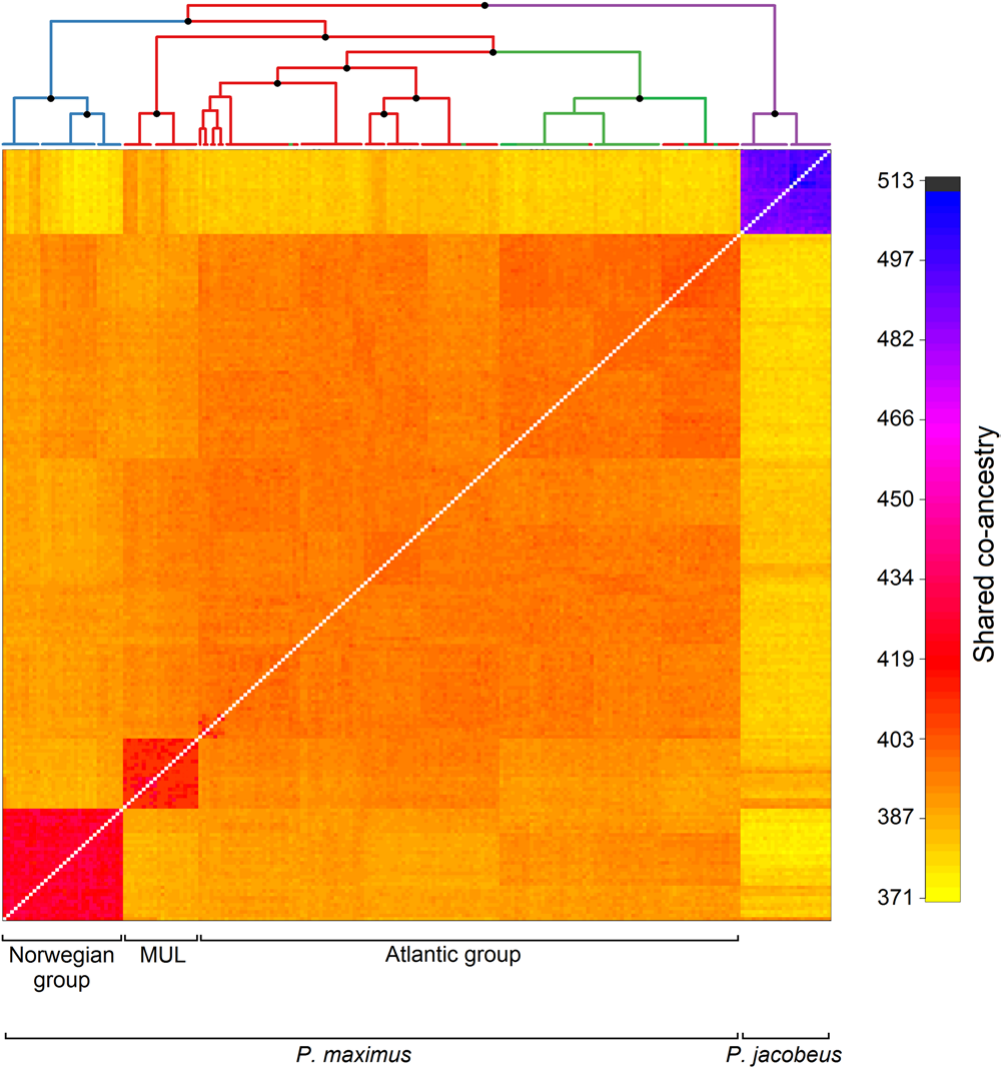
Output of the fineRADstructure analysis of the genomic data. In the cladogram, branches are color coded according to sampling origin as shown in Fig. 1 and nodes with bootstrap support greater than 90% are marked by black points. The heat map depicts variation in pairwise coancestry among individuals according to the scale shown on the right.

Next, we used a model-based Bayesian clustering approach implemented in fineRADstructure^33^ to infer population structure via shared co-ancestry. The resulting co-ancestry matrix and cladogram shown in Fig. 3 largely confirmed the results of the two previous analyses. First, *P. maximus* and *P. jacobeus* were clearly resolved as distinct, well supported clades, with substantially higher levels of shared co-ancestry within *P. jacobeus* reflecting lower levels of genetic diversity (Fig. 3). Second, the Norwegian group clustered apart from the remaining *P. maximus* populations and had slightly higher levels of co-ancestry, as did scallops from Mulroy Bay, which nested within the Atlantic group but were also clearly resolved. Finally, most of the samples from England, Scotland and the Shetland Islands could be readily distinguished from the other Atlantic samples, indicating the possible presence of fine scale population structure.

### Genetic diversity

To explore patterns of genetic diversity, we calculated the number of polymorphic loci (NPL) for each population. In order to standardize across populations with different sample sizes, we randomly sub-sampled five individuals 1,000 times from each population with replacement. We furthermore calculated each individual’s multilocus heterozygosity (MLH). Both measures were significantly higher in *P. maximus* than in *P. jacobeus* (ANOVA, NPL: *F* = 5.44, *p* < 0.05; MLH: *F* = 19.58, *p* < 0.01). Significant differences were also observed between the Atlantic and Norwegian groups of *P. maximus* (ANOVA, NPL: *F* = 13.7, *p* < 0.01; MLH: *F* = 9.24, *p* < 0.01) although the nature of these differences depended on the measure in question. Specifically, NPL was lower in the Norwegian group (Supplementary Fig. 1a) whereas MLH was not (Supplementary Fig. 1b), while MLH also revealed a clear tendency for higher diversity in TND and BAN relative to the other populations (Supplementary Fig. 1b). Finally, in contrast to a previous study based on microsatellites^13^, neither measures of genetic diversity were associated with latitude (NPL: *r*^2^ = 0.03, *p* = 0.54, MLH: *r*^2^ < 0.01, *p* = 0.95), regardless of whether or not TND and BAN were included (NPL: *r*^2^ = 0.03, *p* = 0.6, MLH: *r*^2^ = 0.05, *p* = 0.46).

### Demographic modeling

**Figure 4:**
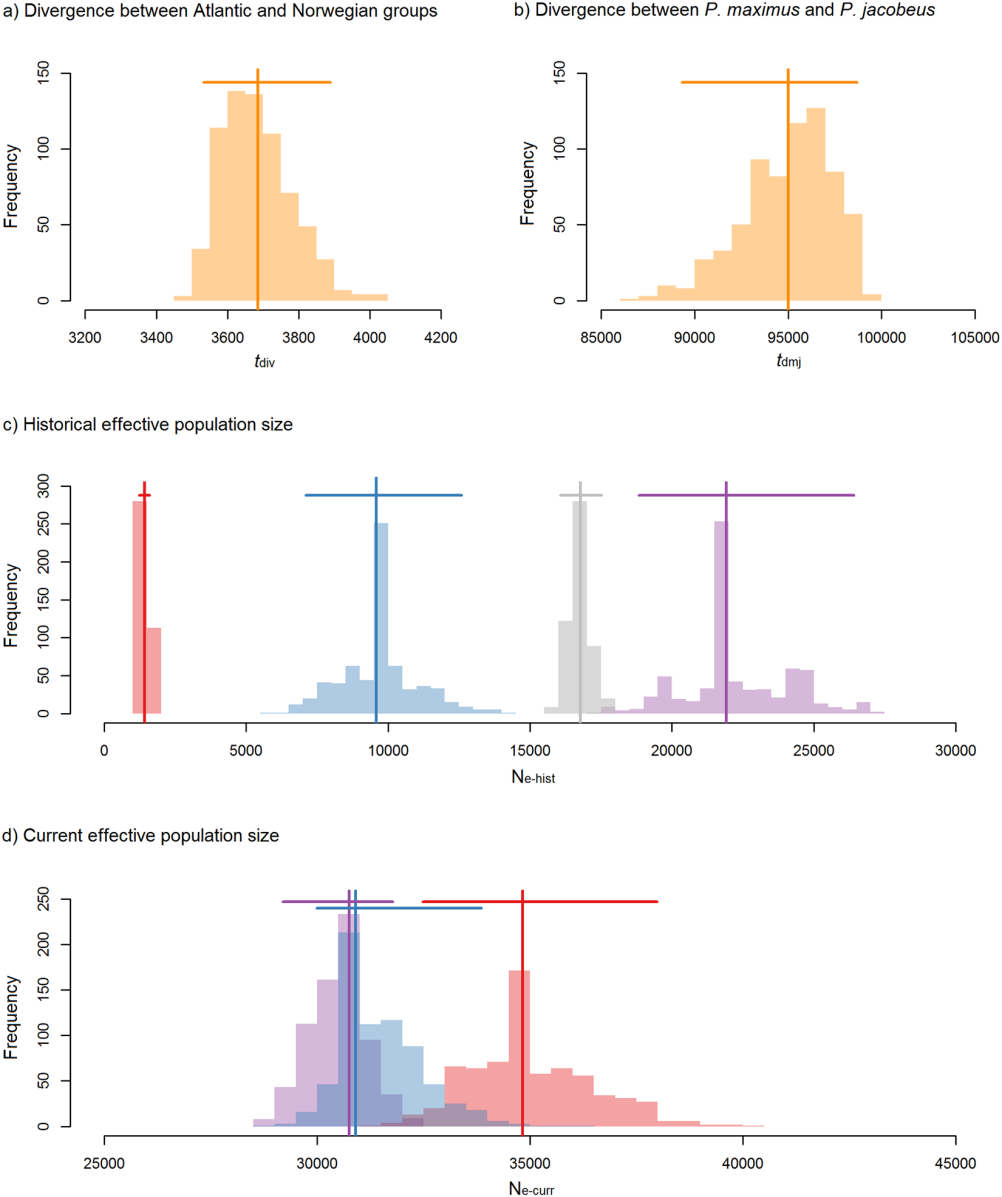
Parameter estimates obtained from the best supported demographic model of *P. maximus* and *P. jacobeus* (see Materials and Methods and Results for details). Estimates from the model are shown as vertical lines together with their associated 95% confidence intervals (horizontal bars) and distributions of parametric bootstrap estimates (shape files). Panel (a) shows the estimated divergence time of *P. maximus* and *P. jacobeus* (in generations ago) and panel (b) shows the estimated divergence time of the Atlantic and Norwegian *P. maximus* groups (in generations ago). Panel (c) shows historical effective population size estimates for all three scallop lineages and panel (d) shows the corresponding contemporary effective population size estimates. In panels (c) and (d), the Atlantic and Norwegian *P. maximus* groups are color coded in red and blue respectively, while *P. jacobeus* is shown in purple. The additional grey colored lineage in panel (c) represents *P. maximus* prior to the divergence of the Atlantic and Norwegian groups.

In order to estimate divergence times and historical effective population size changes of the major European scallop lineages as well as to evaluate support for three alternative demographic models (see Materials and Methods for details), we used the coalescent simulator *fastsimcoal2*^34^ together with the empirical folded site frequency spectra. We found that the model in which the Atlantic and Norwegian *P. maximus* groups originated from two glacial refugia (Supplementary Fig. 2a) received the highest AIC support (Supplementary Table 3). The resulting parameter estimates from the best supported model are shown in Fig. 4 together with their bootstrapped distributions and corresponding 95% confidence intervals (Supplementary Table 4). Within *P. maximus*, the Atlantic and Norwegian groups were estimated to have diverged around 3,700 generations ago (Fig. 4a) and both lineages are inferred to have experienced pronounced expansions (Fig. 4c,d). *P. jacobeus* was estimated to have diverged from *P. maximus* approximately 95,000 generations ago (Fig. 4b) and corresponding estimates of the historical (Fig. 4c) and contemporary effective population sizes of *P. jacobeus* (Fig. 4d) were indicative of modest population expansion in comparison to *P. maximus*. Contemporary effective population size estimates were smallest for *P. jacobeus*, consistent with lower observed levels of genetic diversity (Supplementary Fig. 1), intermediate for the Norwegian *P. maximus* group and largest for the Atlantic *P. maximus* group (Fig. 4d).

### Detection of loci associated with environmental variables

**Figure 5:**
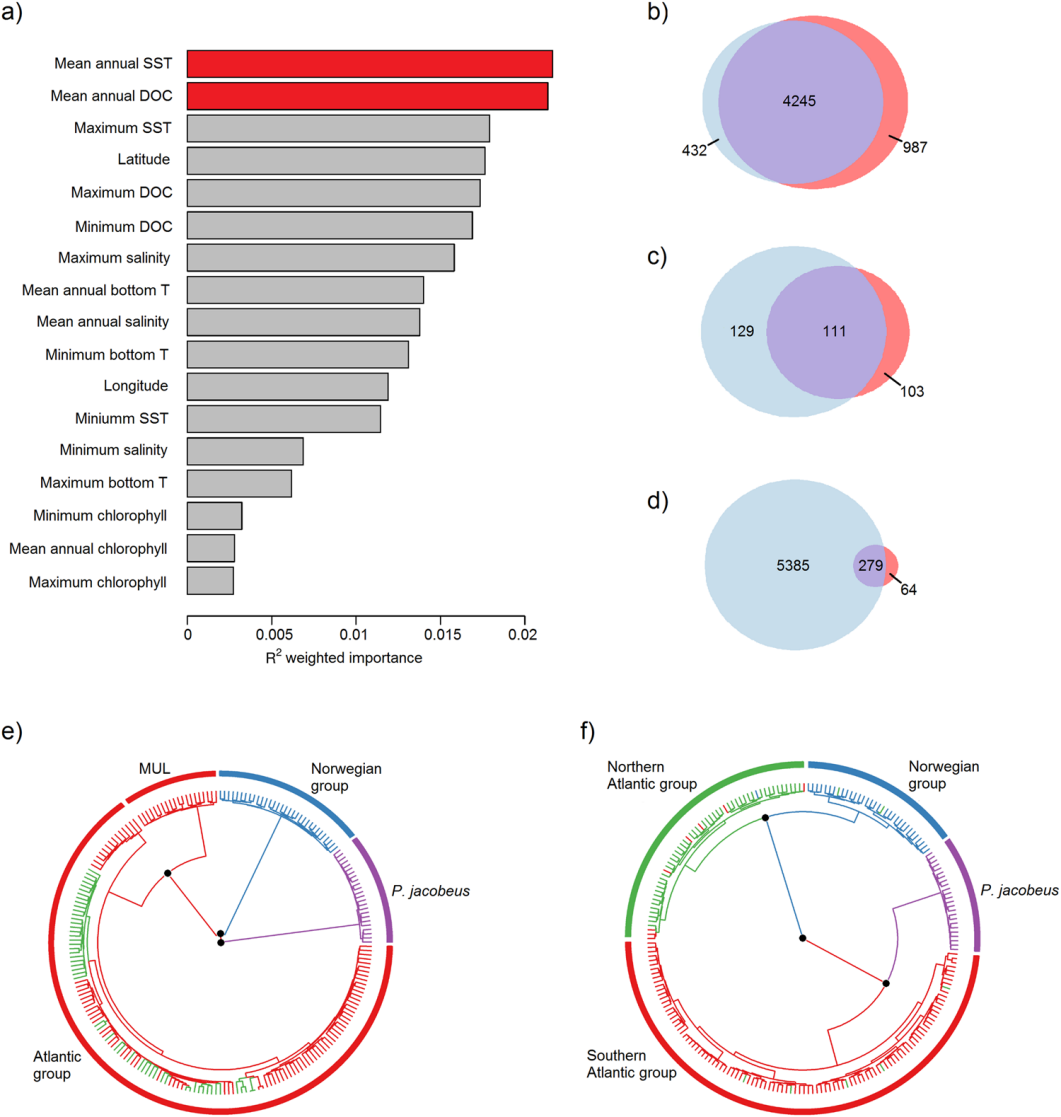
Graphical summary of our analysis of environmentally associated population structure (see Materials and Methods for details). Panel (a) shows the importance of each environmental variable together with latitude and longitude in explaining genetic variation across populations as obtained from the gradient forest analysis. Panel (b) shows the number of loci identified in LFMM analyses of mean annual dissolved oxygen concentration (DOC, light blue) and mean annual sea surface temperature (SST, coral) and their overlap (purple). Panel (c) shows the number of loci identified in BayPass analyses of mean annual dissolved oxygen concentration (DOC, light blue) and mean annual sea surface temperature (SST, coral) and their overlap (purple). Panel (d) shows the total number of candidate loci identified by LFMM (light blue), BayPass (coral) and by both methods (purple). Panels (e) and (f) represent phylogenetic trees constructed from the neutral loci and environmentally associated loci respectively. Tree edges represent individuals and are color coded according to their population of origin as shown in Fig. 1. Nodes with bootstrap support greater than 90% are marked by black points.

We implemented gradient forest analysis using the R package ‘gradientForest’^23^ to understand which environmental factors play a predominant role in explaining genetic variation across European scallop populations. The variables showing the greatest importance were all related to SST and dissolved oxygen concentration (DOC), with mean annual SST and mean annual DOC being the most important (Fig. 5a). We therefore interrogated our dataset to identify loci exhibiting unusual associations with mean annual SST and mean annual DOC. To do so, we employed two different methodologies and considered as candidate environmentally associated loci only those SNPs identified by both methods, with the aim of minimizing false positives.

First, we used latent factor mixed models (LFMMs) within the R package ‘LEA’^35^. After combining results from five independent runs and correcting the resulting *p*-values for the false discovery rate, significant associations were detected at 5,664 loci (6.9%). Of these, 4,245 SNPs were associated with both DOC and SST, 432 SNPs only with DOC and 987 SNPs only with SST (Fig. 5b). Second, we used a Bayesian hierarchical modeling approach implemented in the program BayPass^36^, which recovered strong associations at 343 loci (0.4%). Among them, 111 were associated with both DOC and SST, 129 SNPs only with DOC and 103 SNPs only with SST (Fig. 5c). A total of 279 loci were identified by both methods (Fig. 5d) and were therefore considered as candidate environmentally associated loci.

In order to investigate whether the neutral and environmentally associated loci resolved contrasting phylogenetic patterns, we used the R package ‘ape’^37^ to generate separate phylogenetic trees for both classes of SNP. The neutral phylogeny showed a deep split between *P. jacobeus* and *P. maximus*, the Norwegian group clustered apart from all of the remaining *P. maximus* samples and, within the Atlantic group, only Mulroy Bay was additionally resolved (Fig. 5e). By contrast, the environmentally associated loci revealed a deep and well supported split between scallops from lower and higher latitudes. Notably, the southern and northern Atlantic groups were clearly resolved, with the former being more closely related to *P. jacobeus* and the latter being more closely related to the Norwegian group (Fig. 5f). A similar pattern was obtained when the neutral and environmentally associated datasets were subjected to PCA and fineRADstructure analyses (Supplementary Fig. 3).

## Discussion

We RAD sequenced scallops from across Europe to generate detailed insights into population genetic structure, demographic history and local adaptation. Additionally, we investigated the relationship between *P. maximus* and *P. jacobeus*. Over 80,000 loci were successfully genotyped in the majority of individuals, providing several orders of magnitude greater resolution than previous studies. Our data not only resolved two main lineages within *P. maximus* corresponding to the Atlantic and Norwegian groups defined by Morvezen *et al*.^13^, but also uncovered fine-scale genetic differentiation and identified a suite of loci associated with oceanic oxygen concentration and/or temperature. Furthermore, demographic inference based on coalescent analysis of empirical site frequency spectra suggested that the two *P. maximus* lineages emerged towards the end of the last glacial maximum, whereas *P. jacobeus* diverged from *P. maximus* around 100,000 generations ago, consistent with other genetic studies indicating an earlier than expected split between the two species.

### Genetic differentiation within *P. maximus*

Our results confirm previous studies of *P. maximus* documenting the presence of a Norwegian and an Atlantic group^12,13^. Uncovering the same pattern with a much larger panel of SNPs implies that genetic differences are distributed across the genome, which is consistent with our historical demographic analysis suggesting that the two lineages may have arisen independently from separate glacial refugia (see below). Moreover, by sampling populations from the northern part of the UK and demonstrating that these show greater affinity to the Atlantic group, our study provides a refined picture of the probable location of the boundary between the two groups. Specifically, the lack of appreciable gene flow between Norway and the Shetland Islands is consistent with the Norwegian trench^14^ acting as a barrier to genetic exchange, as has also been observed in several other species of benthic macrofauna^15^.

While Morvezen *et al*.^13^ found evidence for an isolation by distance pattern within the Norwegian group but not within the Atlantic group, our data support the opposite pattern. Specifically, we did not find any genetic differences between the two Norwegian populations included in our study, but analysis of the Atlantic group uncovered multiple significant pairwise *F*_st_ values and both the PCA and fineRADstructure analysis hinted at the possible presence of weak genetic structuring along a north‒south axis. These contrasting outcomes could partly reflect differences in the sampling designs of the two studies. Specifically, our study improved upon Morvezen *et al*.’s^13^ geographic coverage of the Atlantic group by incorporating populations from Ireland, Scotland and the Shetland Islands, but our sampling of the Norwegian group was sparser and did not include the southernmost population of Rennesøy. However, our ability to detect fine-scale population structure within the Atlantic group might also be a consequence of improved genetic resolution, as several recent studies have shown that RAD sequencing has greater power than classical genetic markers to capture subtle population structure^30,38^. Regardless of the exact reason, our results are consistent with knowledge of how temperature affects planktonic larval duration in scallops. The amount of time pelagic larvae spend in the open ocean before settlement is strongly influenced by water temperature, with larvae developing in cold water needing significantly longer to achieve metamorphosis^8^. By implication, scallop larvae may have significantly greater dispersal capabilities at cooler northern latitudes and it is therefore not surprising to see that Norwegian populations display a higher degree of genetic homogeneity. Nevertheless, as larval dispersal can also be influenced by other factors such as larval behavior and oceanography^39^, differences in planktonic larval duration may not be the only mechanism responsible for the observed pattern.

Our results also suggest that Mulroy Bay is the most strongly differentiated population within the Atlantic group. This is consistent with previous studies based on mitochondrial DNA and random amplified fragment polymorphisms that also reported genetic differences between Mulroy Bay and other sites across Europe^9,10,40^ as well as with a more recent ddRAD sequencing study that uncovered significant differences between Mulroy Bay and adjacent populations from along the coast of Northern Ireland^32^. One explanation for this could be that Mulroy Bay is a sea loch with a relatively narrow connection to the open sea that may restrict genetic exchange with other populations. However, great scallops are also commercially bottom cultured at Mulroy Bay and this population has been repeatedly re-stocked following severe population declines. Consequently, even if scallop production is mainly based on wild-caught spat, human intervention could conceivably have led to allele frequency changes relative to wild populations. To investigate how human cultivation practices affect population genetic structure, it would be interesting to conduct a larger population genetic study including several commercially cultured populations.

Our analyses of genetic diversity within *P. maximus* revealed three noteworthy patterns. First, the number of polymorphic loci was lower in the Norwegian group than in the Atlantic group, but mean MLH was not. The former is probably a consequence of the smaller contemporary *N*_e_ of the Norwegian group, while MLH may not show the same pattern because this measure is relatively insensitive to recent variation in *N*_e_ owing to the fact that rare alleles are lost more rapidly from small populations than heterozygosity. A second clearly visible pattern was that MLH was considerably higher in TND and BAN than in the other populations. The most probable explanation for this is that genetic diversity at TND may have been influenced by hatchery-raised scallops released in the wild^41^ while scallops from Mulroy Bay have also been repeatedly imported into the Bantry Bay area^42^. Hence, local population enhancement could potentially have increased average levels of heterozygosity by introducing genetic admixture. However, sampling sizes at these locations were modest and it is possible that the observed excess of heterozygosity may also be caused by a strong family structure. Finally, in contrast to Morvezen *et al*.^13^, who found a significant decrease in microsatellite allelic richness with increasing latitude, we did not find any clear latitudinal patterns in either of the genetic diversity measures. Although the reasons for these contrasting findings remain unclear, one possible contributing factor could be ascertainment bias, as all of the microsatellites genotyped by Morvezen *et al*.^13^ were developed from French samples and may therefore be enriched for polymorphisms that are more abundant in the southern group. Alternatively, as microsatellites and SNPs differ in their mutation rates, it could be possible that these two classes of marker are capturing processes operating over different evolutionary timescales.

### Demographic history of *P. maximus*

Morvezen *et al*.^13^ proposed two alternative hypotheses to explain the origin of the Atlantic and Norwegian groups‒one assuming a single glacial refugium and the other assuming two. The single refugium hypothesis was formulated in light of a decline in microsatellite allele richness with increasing latitude^13^, but we did not observe this pattern in our SNP data. In line with this, our demographic models provided support for the two refugia model. This is arguably not surprising as several northern European benthic marine taxa are known to be structured into deeply divergent and locally restricted genetic clades, suggesting that they probably reoccupied their contemporary ranges from multiple glacial refugia^4^. A number of places in northern Europe have been identified as possible sites of marine glacial refugia including southwest Ireland, Iceland and the Faroe Islands, and the Lofoten Archipelago in northern Norway, although Maggs *et al*.^4^ could not find any clear genetic evidence in support of Norway being an important refugium for benthic marine organisms. However, our results, together with those of a recent study of cockles (*Cerastoderma edule*)^43^, suggest that Norway represents a potential northern refuge for at least two species.

Other aspects of our demographic inference were also consistent with expansion from two glacial refugia. First, we estimated that the Atlantic and Norwegian groups diverged around 3,700 generations ago, which corresponds to around 18,000 years ago assuming a generation time of five years^30^. This timing is consistent with the two lineages having diverged somewhere towards the end of the last glacial maximum (LGM)^44^. Second, historical *N*_e_ estimates were consistently smaller than contemporary ones for both lineages, indicative of parallel postglacial expansions, as also suggested by a previous study^30^. Third, the two groups differed markedly in both their historical and contemporary *N*_e_ estimates. Specifically, the Norwegian group appears to have been historically larger than the southern group, while the contemporary pattern is reversed, indicative of more pronounced postglacial expansion of the Atlantic lineage. This finding is in line with the Atlantic group occupying a considerably larger contemporary geographic range.

### Local adaptation

Despite the expectation that local adaptation should be stronger in species with restricted gene flow^45^, there is a growing consensus that selection can also shape the genetic composition of species with highly dispersive planktonic larvae^17^. In particular, studies of several benthic organisms including the sea scallop *P. magellanicus* have identified ambient temperature as a key selective pressure influencing fine-scale genetic structure^19^. To build upon these studies, we therefore conducted gradient forest analysis to identify the most important explanatory environmental variables, and then sought to characterize candidate environmentally associated loci as those markers identified by both LFMM and BayPass. Around 80% of the loci identified by BayPass were also detected by LFMM, yet only 5% of loci overall were common to both approaches. By implication, BayPass detected a smaller but arguably better supported subset of loci, whereas many of the loci identified by LFMM could potentially be false positives due to the fact that isolation by distance tends to increase the type I error rate in LFMM^46^.

A number of previous studies have detected contrasting phylogenetic patterns at neutral and selected loci, indicative of the widespread presence of environmentally mediated population structure^47–49^. In line with this, we found that SNPs associated with SST and/or DOC revealed markedly different phylogenetic patterns to non-associated loci. Specifically, the southern and northern Atlantic groups were closely related to one another based on the neutral loci, whereas in the environmentally associated phylogeny, the southern Atlantic samples were more closely related to *P. jacobeus* and the northern Atlantic samples were more closely related to the Norwegian group. This pattern clearly reflects the geographical pattern of environmental variation across the European geographical range of the species (Fig. 1).

Our results build upon previous studies of European scallops reporting differences in the growth performance of *P. maximus* populations sampled along a latitudinal cline^20^ as well as proteomic differences between scallops from northern France and Norway^21^. Chauvaud *et al*.^20^ suggested that growth performance could be plastic, without excluding a possible role of genetics. However, at least some traits appear to be under genetic control as a common garden experiment involving Scottish and French scallops did not find changes in reproductive traits in transplanted scallops^22^. Our results are consistent with this notion as they suggest that major European scallop lineages carry genetic adaptations that may influence their physiological performance under different environmental conditions. This is worthy of further exploration because local adaptation could have important implications for the management of scallop populations, particularly in the light of climate change^50^.

### Comparison between *P. maximus* and *P. jacobeus*

The scallops *P. maximus* and *P. jacobeus* have traditionally been thought to constitute separate species on the basis of pronounced morphological differences^51^. However, our data contribute towards a growing realization that *P. maximus* and *P. jacobeus* are less differentiated than would be expected if they had diverged five million years ago^13,28–30^. Moreover, our divergence time estimate was circa 95,000 generations ago, which corresponds to around half a million years assuming a generation time of five years. This is consistent with Rios *et al*.^31^, who concluded that the magnitude of Nei’s *D* at allozymes was not compatible with divergence before 300,000 years ago. It is also in line with Saavedra & Peña^30^, who proposed that the two scallop lineages may have diverged prior to population expansions dated to around 700,000 years ago (95% CI = 300,000‒900,000 years ago) based on mitochondrial DNA. This would imply that *P. maximus* and *P. jacobeus* separated during a period of fluctuating climatic conditions following the end of the Pleistocene, when many bivalve species are known to have become extinct or to have shifted their distributions^52^. Afterwards, genetic differences between these lineages were probably maintained by the Almería-Oran oceanographic front (AOF)^53^, which is known to constitute an impassable barrier to gene flow in scallops^31^.

Intriguingly, although our demographic analysis suggested that *P. jacobeus* may have had a larger historical effective population size than *P. maximus*, contemporary estimates point towards a contrasting pattern that is more consistent with the observed levels of genetic diversity. In particular, the contemporary *N*_e_ of *P. jacobeus* appears to be only slightly larger than the historical one, implying relatively limited population growth, while it is also considerably lower than equivalent estimates for the Atlantic and Norwegian *P. maximus* groups. Overall, there is a clear correspondence between contemporary *N*_e_ estimates and levels of genetic diversity across all three major European scallop lineages, providing a plausible explanation for the lower genetic diversity of *P. jacobeus* and suggesting that a significant fraction of the genetic diversity of *P. maximus* may have built up during concerted population expansions that followed LGM.

## Materials and Methods

### Sample collection

A total of 280 scallop samples were collected from 14 populations around Europe (Fig. 1 and Table 1). Twelve of these populations were from the Atlantic coast of Europe (*P. maximus*) and two were from the Mediterranean Sea (*P. jacobeus*). All of these populations are naturally occurring, with the exception of Mulroy Bay (MUL), where scallops are cultured from locally sourced broodstock. Samples from Bantry Bay (BAN) were collected from an area where scallops from Mulroy Bay have been repeatedly imported^42^ and one of the two populations sampled from Brittany (TND) may also be influenced by hatchery-reared scallops, as the Tinduff hatchery (Bay of Brest, Finistère, France) has repeatedly released individuals into the wild as part of a seeding program aimed at increasing scallop population size following decreases due to overfishing^41^. Adductor muscle tissue was taken from 20 adult scallops per location and stored individually in 95% ethanol at −20 °C.

### DNA extraction, RAD sequencing and bioinformatics analysis

Whole genomic DNA was extracted from each sample using an adapted phenol-chloroform protocol^54^ and shipped to the Beijing Genomics Institute for RAD sequencing^55^. Libraries were constructed using the restriction enzyme PstI and sequenced on an Illumina HiSeq. 4000. Quality of the demultiplexed sequence reads was assessed using FastQC (http://www.bioinformatics.babraham.ac.uk/projects/fastqc/) and 17 samples (6%) that generated substantially fewer reads (<750,000, Supplementary Fig. 4) were excluded from further analyses.

The resulting dataset was *de novo* assembled using the Stacks 2.1 pipeline^56^. Values of the three main parameters –m, –M and –n were chosen following the optimization procedure described by Rochette & Catchen^57^. Briefly, –m was set to three and a range of values for –M and –n were evaluated. The combination of these parameters yielding the highest number of polymorphic loci present in at least 80% of the individuals was then defined as optimal. Two different strategies were employed: –n was either set as equal to –M or one unit greater, to account for the potential presence of polymorphisms that are fixed in either *P. maximus* or *P. jacobeus* samples^58^. The optimal combination (m = 3, M = 4 and n = 4; Supplementary Fig. 5) was selected for analyzing the entire dataset. However, only the 100 samples with the greatest depth of coverage were used to generate the catalog to decrease potential noise^57^. The raw genotypes were subsequently filtered to retain only biallelic SNPs (no indels) with both genotype quality and depth of coverage greater than 5 using VCFTools^59^, as well as to retain only SNPs genotyped in at least 80% of the individuals. Next, we discarded all SNPs with a depth of coverage greater than twice the mean depth of coverage of the raw SNP dataset (>28) to remove potentially paralogous loci. Subsequently, all individuals with more than 20% missing data were removed and only variants with MAF greater than 0.01 were retained. Finally, the software PLINK (version 1.9)^60^ was used to eliminate SNPs showing significant departures from Hardy-Weinberg equilibrium with an alpha level of 0.05, as well as to prune out putatively linked loci using an *r*^2^ threshold of 0.5. Detailed information on how the SNPs were filtered and on the number of SNPs that were retained after each filtering step are provided in Supplementary Table 1.

### Genetic structure

Three complimentary analyses were conducted to characterize the strength and pattern of genetic structure within our dataset. First, we calculated pairwise *F*_ST_ values among all of the sampling localities and determined statistical significance with 1,000 permutations using the software Arlequin version 3.5.2.2^61^. Table-wide Bonferroni correction was then applied to account for multiple testing, which resulted in a corrected alpha level of 0.0006. Second, we subjected the data to principal component analysis (PCA) within the R package Adegenet version 2.1.1^62^. Third, we used fineRADstructure^33^ to infer genetic structure using a model-based Bayesian clustering approach that groups together individuals with high levels of shared coancestry. This program is a modified version of the finestructure program^63^ that does not require information on either chromosomal locations or phase of the markers. A “coancestry matrix”, defined as a summary of nearest neighbor haplotype relationships, is required as input and was generated using the ‘RADpainter’ module of fineRADstructure. We subsequently used the default parameters of 100,000 Markov chain Monte Carlo (MCMC) iterations with a burn-in of 100,000 iterations and sampling occurring every 1,000 iterations to run the analysis. Finally, a tree was constructed with 10,000 hill-climbing iterations and the results were visualized using the scripts FINERADSTRUCTUREPLOT.R and FINESTRUCTURELIBRARY.R, which are available via http://cichlid.gurdon.cam.ac.uk/fineRADstructure.html.

### Genetic diversity

We next explored patterns of genetic diversity by calculating two summary statistics for each population. First, we derived the number of polymorphic loci (NPL) after correcting for sample size variation using a randomization approach. Specifically, we extracted subsamples of five individuals from each population 1,000 times with replacement and calculated the number of polymorphic loci in each subsample. Five samples were extracted from each population because this corresponds to the lowest sample size in our dataset (Table 1). Second, we calculated individual multilocus heterozygosity (MLH) using the R package InbreedR^64^.

### Demographic modeling

To estimate divergence times of the main European scallop lineages and to evaluate historical effective population size changes, we implemented demographic analysis using the coalescent simulator *fastsimcoal2*^34^ in combination with the folded site frequency spectra (SFS) calculated from our data using the program Arlequin version 3.5.2.2. As Arlequin cannot incorporate loci with missing genotypes into SFS calculations, we used a subset of 92 individuals genotyped at 15,712 SNPs comprising 22 *P. jacobeus*, 50 *P. maximus* from the Atlantic group and 20 *P. maximus* from the Norwegian group. The aims of this analysis were to estimate the divergence time of *P. maximus* and *P. jacobeus* and to investigate the origin of the Atlantic and Norwegian *P. maximus* groups by formally testing two alternative hypotheses proposed by Morvezen *et al*.^13^, one assuming expansion from a single glacial refugium and the other assuming that the two groups originated from separate glacial refugia.

In order to test these two hypotheses, we evaluated support for two alternative demographic models. The first model (Supplementary Fig. 2a) specified two lineages, one corresponding to *P. jacobeus* and the second to *P. maximus*, which diverged *t*_dmj_ generations ago. Subsequently, at *t*_div_ generations ago, the *P. maximus* lineage split into the Atlantic and Norwegian groups as a result of two independent recolonization events originating from separate glacial refugia. To account for historical population expansion in both *P. maximus* and *P. jacobeus*^30^, we assumed population expansion for all three demes. The second model (Supplementary Fig. 2b) was identical with the exception that the *P. maximus* lineage leading to the Atlantic and Norwegian groups originated from a single refugium, from which it began to expand *t*_exp_ generations ago. Subsequently, the Norwegian group emerged *t*_div_ generations ago as a result of founder effect. Finally, in order to account for the fact that a geographic expansion can sometimes leave a signal of demographic bottleneck^65^, we constructed a third model (Supplementary Fig. 2c) in which no population expansion was assumed between *t*_exp_ and *t*_div_ , and *t*_div_ was assumed to be more recent. For all three models, in addition to *t*_div_ and *t*_exp_, we estimated historical and contemporary effective population sizes for *P. jacobeus* (N_e-hist-JAC_ and N_e-cur-JAC_), the historical effective population size of the *P. maximus* lineage that later split to form the Atlantic and Norwegian groups (N_e-hist-MAX_), and the historical and contemporary effective population sizes of the Atlantic (N_e-hist-ATL_ and N_e-cur-ATL_) and Norwegian groups (N_e-hist-NOR_ and N_e-cur-NOR_). All priors for these analyses are given in Supplementary Table 5.

For each model, we performed 50 independent *fastsimcoal2* runs with 100,000 simulations and 40 cycles of the likelihood maximization algorithm. The run yielding the highest maximum likelihood value for each model was then selected to calculate Akaike’s Information Criteria (AIC) as described by Varin & Vidoni^66^ to allow model comparison. We then extracted parameter estimates from the best run of the model receiving highest support together with their 95% confidence intervals obtained via parametric bootstrapping as described in Excoffier *et al*.^34^. Specifically, we simulated 100 folded site frequency spectra based on the parameters estimated from the best model and used them to re-estimate models parameters following the same procedure described above.

### Detection of loci associated with environmental variables

We extracted from the Copernicus Marine Service Information (http://marine.copernicus.eu) data for chlorophyll concentration, dissolved oxygen concentration, salinity, sea bottom temperature and sea surface temperature for each of the 14 sampled locations. We then computed minimum, maximum and mean annual values for each of these environmental factors to obtain a total of 18 environmental variables. We then tested which of these environmental variables best explained genetic variation among the sampled scallop populations by using gradient forest analysis as implemented in the R package gradientForest^23^. For this analysis, in addition to the 18 environmental variables, we included latitude and longitude information to assess the importance of geography. Gradient forest is a machine learning regression tree based algorithm that searches for breaks across the environmental predictors. This method was originally implemented to study species turnover in community ecology datasets and was later adapted to detect allelic turnover in genomic data^24^. Gradient forest provides a ranked list of the relative predictive power of all environmental variables and therefore makes it possible to identify those environmental variables that best explain genetic variation.

We then employed the R package LEA^35^ and the program BayPass^36^ to search for associations between individual SNPs and those environmental variables with the greatest predictive power. The first approach uses latent factor mixed models (LFMMs) to detect loci showing unusual associations with environmental variables compared to the genomic background. It accounts for the underlying population structure by introducing latent factors while simultaneously estimating random effects due to population history and isolation by distance. We ran 10,000 iterations of the Gibbs sampling algorithm with the first 5,000 iterations discarded as burn-in. *Z* scores from five independent runs were then combined and the resulting *p*-values were corrected for the false discovery rate according to Benjamini & Hochberg^67^ with an alpha level of 0.01. The second approach uses a Bayesian hierarchical model approach to identify loci associated with environmental variables, while explicitly accounting for the neutral covariance structure across population allele frequencies by incorporating a covariance matrix of population allele frequencies resulting from their shared history. We ran BayPass using default parameters under the standard covariate model and identified loci associated with environmental variables as those showing a Bayes Factor greater than 10, which is indicative of strong evidence^36^.

## Supplementary information

Supplementary Information

## Data Availability

The raw sequence reads used to generate the results of this study are available at the Short Read Archive (SRA Accession: PRJNA526674).
