# Supplementary material for: RAD sequencing sheds new light on the genetic structure and local adaptation of European scallops and resolves their demographic histories
Source: Sci Rep. 2019 May 15;9:7455. doi: 10.1038/s41598-019-43939-4 (PMC6520335; doi:10.1038/s41598-019-43939-4)
Supplement: Supplementary file 1 — Supplementary Information [file 41598_2019_43939_MOESM1_ESM.pdf]

RAD sequencing sheds new light on the genetic structure and local adaptation of European scallops and resolves their demographic histories.

David L. J. Vendrami, Michele De Noia, Luca Telesca, William Handal, Grégory Charrier, Pierre Boudry, Luke Eberhart-Philips, Joseph I. Hoffman

Supplementary Information

Supplementary materials

Supplementary Table 1: Table showing the different filtering steps that were carried out to generate the final high-quality SNP dataset starting from the raw SNPs output by the Stacks pipeline. The number of retained SNPs after each step is reported.

Filtering step	Number of retained SNPs	Notes
Raw biallelic SNPs	564,526	-
Genotype depth of coverage > 5	564,526	During this step SNPs were not discarded. Instead single genotypes with depth of coverage equal or smaller than 5 were replaced with a NA.
Genotype quality > 5	564,526	During this step SNPs were not discarded. Instead single genotypes with genotype quality equal or smaller than 5 were replaced with a NA.
SNP genotyping rate > 80%	306,490	Only SNPs genotyped in at least 80% of individuals were retained.
Depth of coverage < 28	302,298	SNPs with depth of coverage greater than 28 (twice the mean depth of coverage of the raw dataset) were removed.
Individual genotyping rate > 80%	302,298	At this stage only individuals genotyped at least 80% of the loci were retained (61 samples were removed).
MAF > 0.01	126,737	Only SNPs with MAF greater than 0.01 were retained.
Hardy-Weinberg equilibrium p-value > 0.05	93,610	All loci that showed significant (p < 0.05) departure from HWE were removed. HWE p-values were calculated in PLINK specifying the --midp flag following PLINK recommendations.
Linkage Disequilibrium pruning: r2 < 0.5	82,439	SNP located within the same RAD tag showing a r2 value greater than 0.5 were removed.
Final quality-filtered SNP dataset	82,439	-

Supplementary Table 2: Table showing pairwise F_{ST} values (below diagonal) and corresponding *p*-values (above diagonal) for pairwise population comparisons. Values highlighted in bold were significant after table-wide Bonferroni correction for multiple tests.

	MUL	ADR	OBN	PLY	RDB	RDA	MPJ
MUL	0	0.000+-0.0000	0.000+-0.0000	0.000+-0.0000	0.000+-0.0000	0.000+-0.0000	0.000+-0.0000
ADR	**0.06466**	0	0.000+-0.0000	0.00010+-0.0001	0.00010+-0.0001	0.00010+-0.0001	0.00020+-0.0001
OBN	**0.02316**	**0.05523**	0	0.000+-0.0000	0.000+-0.0000	0.000+-0.0000	0.000+-0.0000
PLY	**0.02419**	**0.05853**	**0.00236**	0	0.39636+-0.0006	0.000+-0.0000	0.000+-0.0000
RDB	**0.02068**	**0.05588**	**0.00029**	-0.00168	0	0.000+-0.0000	0.000+-0.0000
RDA	**0.01924**	**0.0531**	**0.0065**	**0.0062**	**0.00225**	0	0.000+-0.0000
MPJ	**0.05498**	**0.00458**	**0.04852**	**0.04917**	**0.04462**	**0.04271**	0
SHE	**0.02027**	**0.06157**	-0.0073	-0.00519	-0.0039	**0.00553**	**0.04405**
NBG	**0.04557**	**0.08547**	**0.02331**	**0.02818**	**0.02796**	**0.03257**	**0.06649**
NFB	**0.04848**	**0.08388**	**0.02652**	**0.03184**	**0.03082**	**0.0334**	**0.06784**
RLB	**0.01974**	**0.05146**	**0.00431**	**0.00213**	-0.00125	-0.0003	**0.044**
PEB	**0.01892**	**0.05826**	**0.00213**	**0.00239**	**0.00111**	**0.00252**	**0.0455**
BAN	**0.00541**	**0.05239**	-0.00927	-0.00439	-0.00481	-0.0098	**0.03418**
TND	**0.0176**	**0.05901**	-0.00077	**0.00029**	-0.00052	**0.00011**	**0.043**

	SHE	NBG	NFB	RLB	PEB	BAN	TND
MUL	0.000+-0.0000	0.000+-0.0000	0.000+-0.0000	0.000+-0.0000	0.000+-0.0000	0.000+-0.0000	0.000+-0.0000
ADR	0.000+-0.0000	0.000+-0.0000	0.00010+-0.0001	0.00010+-0.0001	0.000+-0.0000	0.000+-0.0000	0.00010+-0.0001
OBN	0.99980+-0.0001	0.000+-0.0000	0.000+-0.0000	0.000+-0.0000	0.000+-0.0000	0.85298+-0.0031	0.67379+-0.0008
PLY	0.99455+-0.0007	0.000+-0.0000	0.000+-0.0000	0.000+-0.0000	0.000+-0.0000	0.58412+-0.0032	0.00010+-0.0001
RDB	0.51926+-0.0051	0.000+-0.0000	0.000+-0.0000	0.07970+-0.0029	0.000+-0.0000	0.60248+-0.0012	0.12458+-0.0011
RDA	0.000+-0.0000	0.000+-0.0000	0.000+-0.0000	0.30251+-0.0021	0.00000+-0.0000	0.24803+-0.0005	0.00010+-0.0001
MPJ	0.00000+-0.0000	0.00000+-0.0000	0.00000+-0.0000	0.00000+-0.0000	0.00000+-0.0000	0.00000+-0.0000	0.00000+-0.0000
SHE	0	0.00000+-0.0000	0.00000+-0.0000	0.32472+-0.0019	0.00000+-0.0000	0.46755+-0.0031	0.00000+-0.0000
NBG	**0.02953**	0	0.55311+-0.0054	0.00000+-0.0000	0.00000+-0.0000	0.00000+-0.0000	0.00000+-0.0000
NFB	**0.0303**	-0.00484	0	0.00000+-0.0000	0.00000+-0.0000	0.00000+-0.0000	0.00000+-0.0000
RLB	-0.00173	**0.0273**	**0.03002**	0	0.40116+-0.0002	0.97515+-0.0016	0.19216+-0.0044
PEB	**0.00207**	**0.03225**	**0.0339**	-0.00041	0	0.21168+-0.0012	0.000+-0.0000
BAN	-0.00132	**0.02835**	**0.02838**	-0.01157	-0.00738	0	0.42251+-0.0011
TND	**0.00332**	**0.03366**	**0.03495**	-0.00334	**0.0003**	-0.00137	0

Supplementary Table 3: Relative maximum ln(likelihoods), number of estimated parameters and AIC values for the three alternative demographic models. Maximum ln(likelihood) values are given as the best value among the 50 independent runs for each model.

model	max ln(likelihood)	No. of parameters	AIC	Model normalized relative likelihood (w)
2 refugia	-234,438.10	9	468,894.20	1
1 refugium with expansion	-245,144.70	10	490,309.40	0
1 refugium without expansion	-246,559.70	10	493,139.40	0

Supplementary Table 4: Estimated parameter values and their associated 95% confidence intervals from the best supported demographic model of *P. maximus* and *P. jacobeus*. See the Materials and Methods for descriptions of the estimated parameters.

Parameter	Estimate	95% CI
$N_{e\text{-}hist\text{-}ATL}$	1,413	1,233; 1,591
$N_{e\text{-}hist\text{-}NOR}$	9,581	7,099; 12,596
$N_{e\text{-}cur\text{-}ATL}$	34,825	32,481; 37,983
$N_{e\text{-}cur\text{-}NOR}$	30,901	29,984; 33,860
T_{div}	3,685	3,531; 3,888
$N_{e\text{-}hist\text{-}JAC}$	21,923	18,824; 26,414
$N_{e\text{-}hist\text{-}MAX}$	16,773	16,063; 17,517
$N_{e\text{-}cur\text{-}JAC}$	30,755	29,190; 37,778
T_{dmj}	95,004	89,318; 98,698

Supplementary Table 5: Table showing the priors for all estimated parameter separately for each demographic model.

Parameter	Priors in "Two refugia" model	Priors in "One refugium with expansion" model	Priors in "One refugium without expansion" model
$N_{e\text{-hist-ATL}}$	10; 10,000	10; 10,000	10; 10,000
$N_{e\text{-hist-NOR}}$	10; 10,000	10; 10,000	10; 10,000
$N_{e\text{-cur-ATL}}$	100; 100,000	100; 100,000	100; 100,000
$N_{e\text{-cur-NOR}}$	100; 100,000	100; 100,000	100; 100,000
T_{div}	3,000; 10,000	3,000; 10,000	1,000; 5,000
$N_{e\text{-hist-JAC}}$	100; 100,000	100; 100,000	100; 100,000
$N_{e\text{-hist-MAX}}$	100; 100,000	100; 100,000	100; 100,000
$N_{e\text{-cur-JAC}}$	100; 100,000	100; 100,000	100; 100,000
T_{dmj}	50,000; 300,000	50,000; 300,000	50,000; 300,000
T_{exp}	-	3,000; 10,000	3,000; 10,000

a)

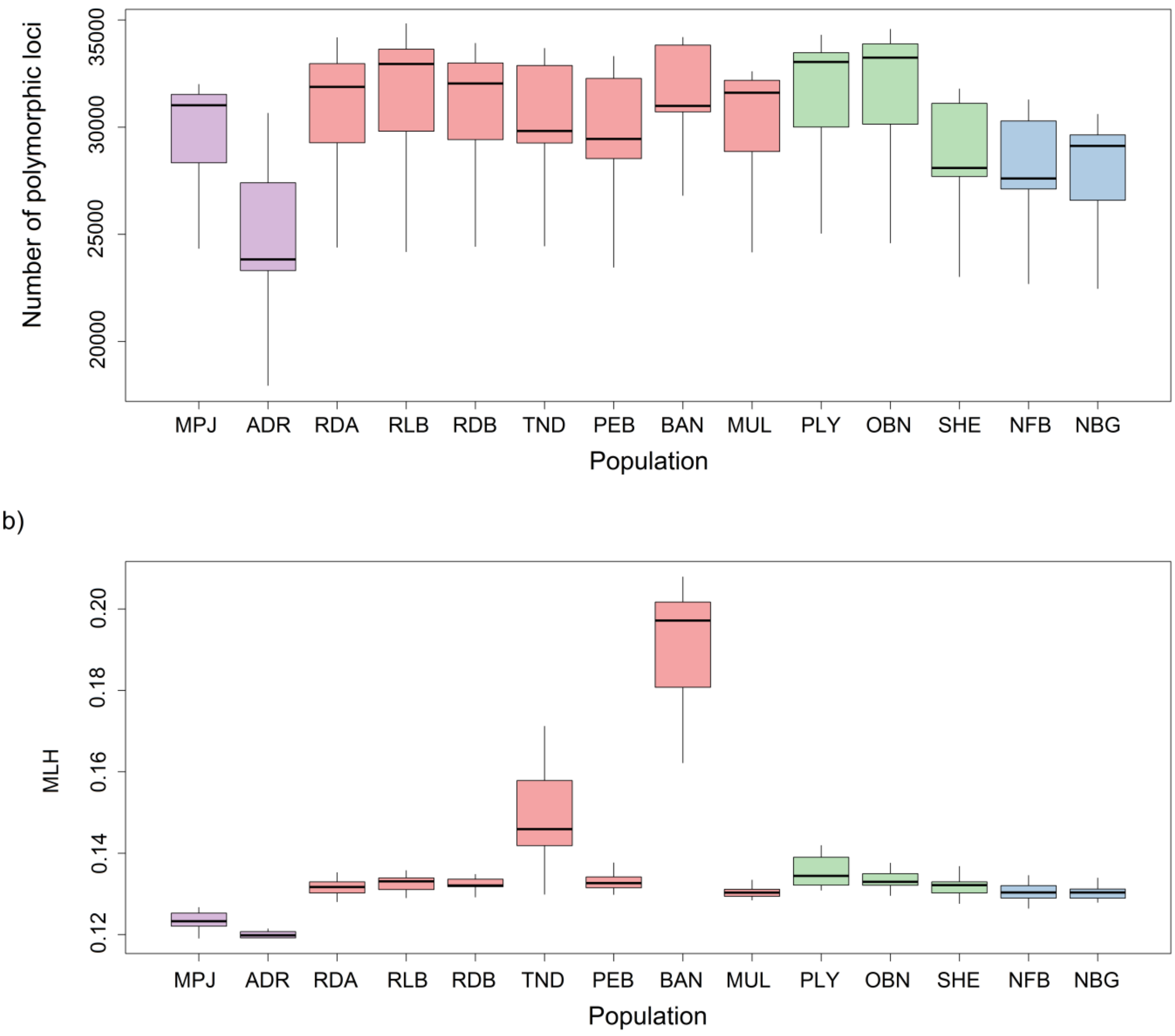

Supplementary Figure 1: Variation in genetic diversity, quantified as (a) the number of polymorphic loci (NPL) and (b) multilocus heterozygosity (MLH). The data are represented using standard Tukey boxplots with each population color coded as shown in Figure 1.

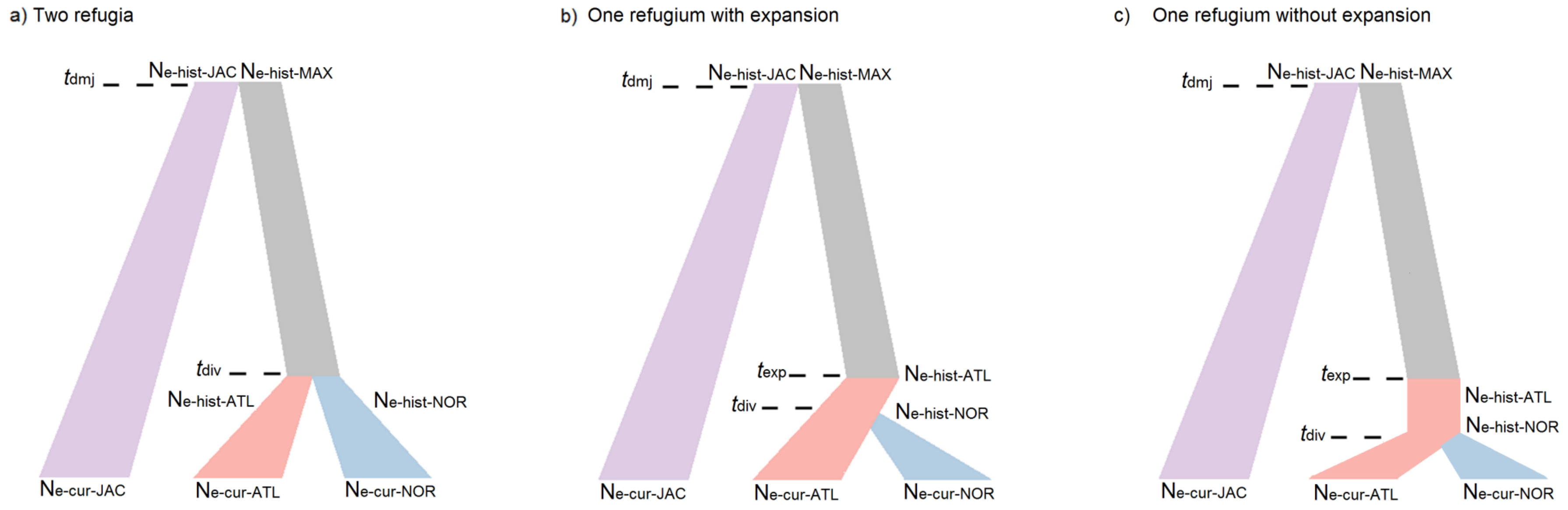

Supplementary Figure 2: Schematic showing tested alternative demographic models. In all three models, the *P. maximus* (grey) and *P. jacobeus* (purple) lineages diverged t_{dmj} generations ago. Subsequently, model (a) assumes that the Atlantic (red) and Norwegian (blue) groups diverged t_{div} generations ago as a result of two independent recolonization events originating from two glacial refugia, whereas model (b) assumes that t_{exp} generations ago the recolonization of Europe started from a single refugia in Southern Europe and that t_{div} generations ago the Norwegian group emerged as a consequence of founder effect. Model (c) was similar to model (b) but assumed no population expansion between t_{exp} and t_{div}, and a more recent t_{div}. Historical and contemporary effective population sizes are indicated as $N_{e\text{-hist-JAC}}$, $N_{e\text{-cur-JAC}}$ for *P. jacobeus*, as $N_{e\text{-hist-ATL}}$, $N_{e\text{-cur-ATL}}$ for the *P. maximus* Atlantic group and as $N_{e\text{-hist-ATL}}$, $N_{e\text{-cur-ATL}}$ for the *P. maximus* Norwegian group.

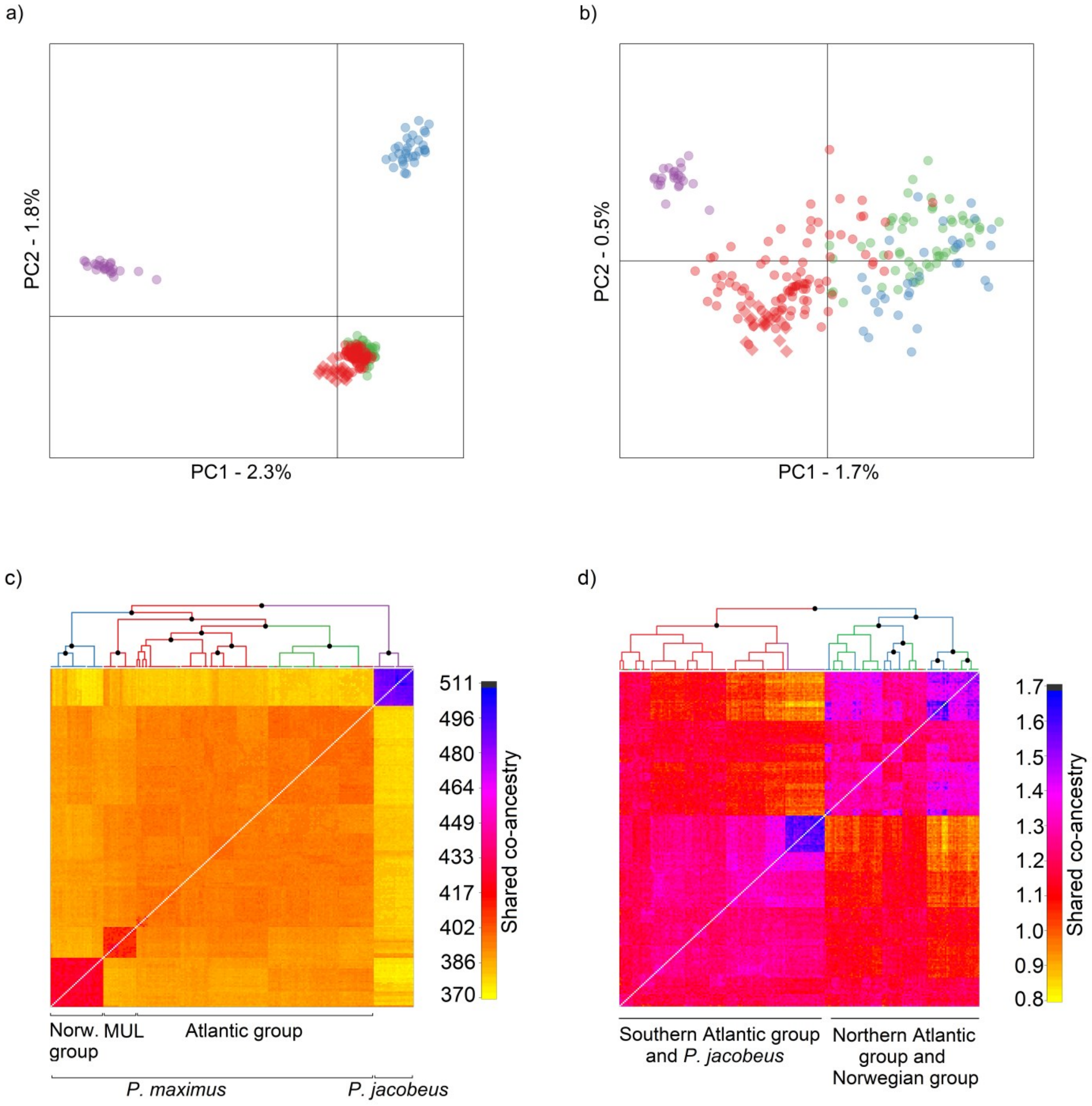

Supplementary Figure 3: Scatterplot showing individual variation in principal component (PC) scores derived from principal component analysis (a and b) and fineRADstructure outputs (c and d) carried out separately for the neutral loci (a and c) and the environmentally-associated loci (b and d). In panels (a) and (b), samples are color coded as described in the legend of Figure 1 and scallops from Mulroy Bay are indicated by diamonds. In panels (c) and (d), the branches of the cladogram are color coded according to sampling origin as shown in Figure 1 and nodes with bootstrap support greater than 90% are marked by black points.

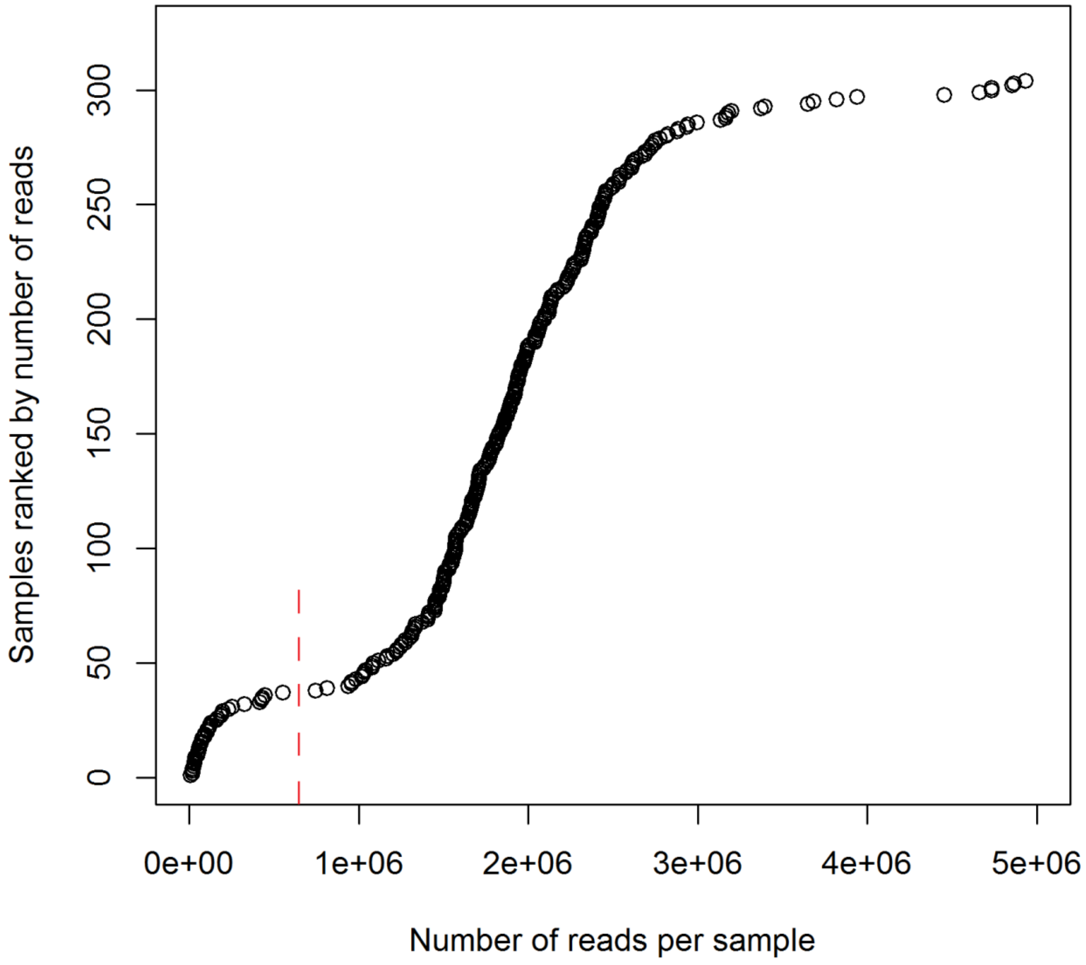

Supplementary Figure 4: Distribution of the number of raw reads obtained for each sample, ranked in ascending order. The dashed red line shows the number of reads below which samples were discarded.

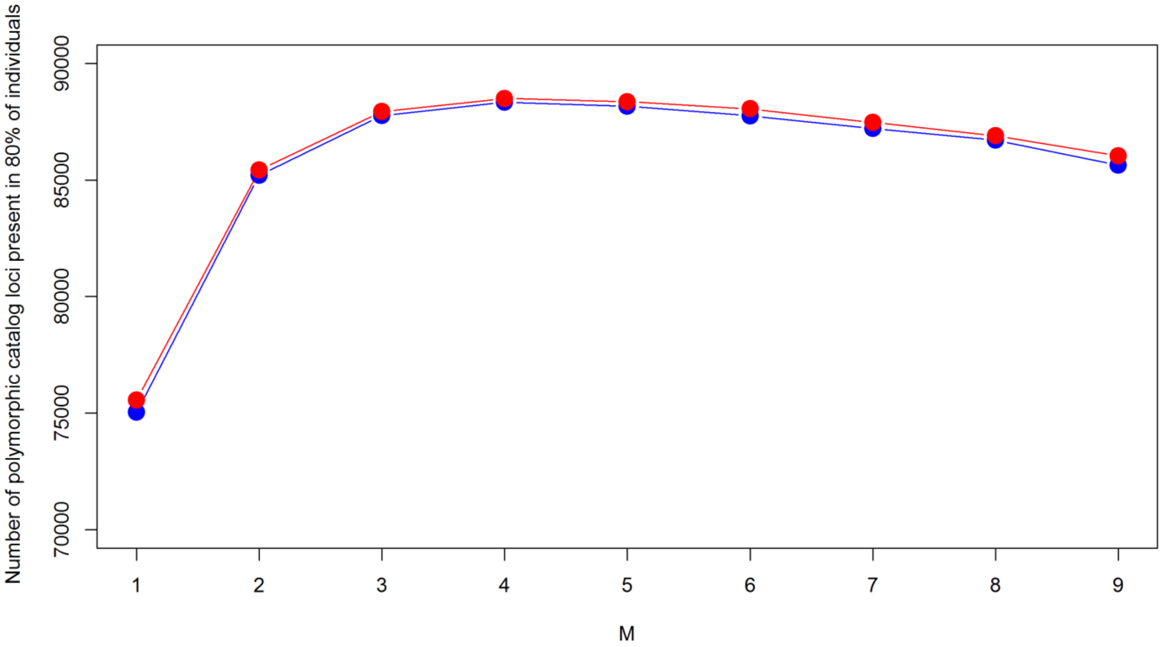

Supplementary Figure 5: Change in the number of polymorphic *de novo* assembled loci present in at least 80% of the samples for increasing values of the –M parameter (see Materials and Methods for details). The red and the blue lines correspond to assemblies performed by setting –n = –M and –n = –M + 1 respectively. At –M = 4, the number of assembled loci reached its maximum value and this value was therefore selected for the analysis of the full dataset. –n = 4 was chosen over –n = 5 because it yielded a greater total number of loci.
